# Systematic review and meta-analysis of nasal potential difference in hypoxia-induced lung injury

**DOI:** 10.1038/srep30780

**Published:** 2016-08-04

**Authors:** Zhenlei Su, Lili Zhu, Jing Wu, Runzhen Zhao, Hong-Long Ji

**Affiliations:** 1 Institute of Lung and Molecular Therapy, Xinxiang Medical University, Xinxiang Henan 453003, China; 2 School of Public Health, Xinxiang Medical University, Xinxiang Henan, 453003, China; 3 School of Nursing, Xinxiang Medical University, Xinxiang Henan 453003, China; 4 Department of Cellular and Molecular Biology, University of Texas Health Science Center at Tyler, Tyler, Texas, 75708, USA; 5 Texas Lung Injury Institute, University of Texas Health Science Center at Tyler, Tyler, Texas, 75708, USA

## Abstract

Nasal potential difference (NPD), a well-established *in vivo* clinical test for cystic fibrosis, reflects transepithelial cation and anion transport in the respiratory epithelium. To analyze whether NPD can be applied to diagnose hypoxic lung injury, we searched PubMed, EMBASE, Scopus, Web of Science, Ovid MEDLINE, and Google Scholar, and analyzed data retrieved from eleven unbiased studies for high altitude pulmonary edema (HAPE) and respiratory distress syndrome (RDS) using the software RevMan and R. There was a significant reduction in overall basal (WMD −5.27 mV, 95% CI: −6.03 to −4.52, P < 0.00001, I^2^ = 42%), amiloride-sensitive (ENaC) (−2.87 mV, 95% CI: −4.02 to −1.72, P < 0.00001, I^2^ = 51%), and -resistant fractions (−3.91 mV, 95% CI: −7.64 to −0.18, P = 0.04, I^2^ = 95%) in lung injury patients. Further analysis of HAPE and RDS separately corroborated these observations. Moreover, SpO_2_ correlated with ENaC-associated NPD positively in patients only, but apparently related to CFTR-contributed NPD level inversely. These correlations were confirmed by the opposite associations between NPD values and altitude, which had a negative regression with SpO_2_ level. Basal NPD was significantly associated with amiloride-resistant but not ENaC fraction. Our analyses demonstrate that acute lung injury associated with systemic hypoxia is characterized by dysfunctional NPD.

Acute lung injury (ALI) could be caused by systemic hypoxia subsequent to sepsis and high altitude^1^. Both high altitude pulmonary edema (HAPE) and acute respiratory distress syndrome (ARDS) are characterized by dysfunctional blood-gas barrier. Common symptoms of ALI include shortness in breathing, coughing, increased respiratory rate and heart beating, cyanosis, and fatigue. Two classic hallmarks of ALI, as revealed by laboratory tests, are pulmonary edema and reduced peripheral capillary oxygen saturation (SpO_2_). The balance of fluid turnover and re-absorption across the epithelial layer is finely maintained by the machinery of membrane protein assemble, including apically located ion channels (i.e., epithelial sodium channels-ENaC, cystic fibrosis transmembrane conductance regulator-CFTR) and basolateral Na^+^/K^+^-ATPase. This vectorial transepithelial process is rate-limiting and sensitive to oxygen level^2^. In addition, hypoxia led to increased microvascular pressure and transendothelial permeability to flood the air spaces^3^,^4^,^5^,^6^,^7^.

Injury of the epithelial and endothelial layers of the blood-gas barrier could be tested by *in vivo* alveolar fluid clearance (AFC) and labeled albumin in collected edema fluid specimens, respectively^2^. In comparison, measurements of nasal potential difference (NPD) are able to identify damaged apical membrane proteins functionally in a fast on-site manner^8^. This measure is easy to be carried out at both clinic and bedside and well-tolerated by patients^9^,^10^,^11^,^12^,^13^,^14^. This technique has been applied as a routine test for cystic fibrosis to detect abnormal nasal transepithelial potential difference resulted from transepithelial movement of positive and negative charges via hyperactive ENaC and deficient CFTR, respectively. Given that several similar features in epithelial morphology and bioelectric properties are documented between the nasal, the airway, and the alveolar epithelium in mammal, NPD has been applied to ALI and other lung diseases to reflect the transalveolar ion transport activity^15^. The total or basal NPD was composed of electrogenic transepithelial Na^+^ and Cl^−^ movement-generated ion gradients. To distinguish them, either amiloride, a specific blocker of ENaC, or Na^+^-free saline was used to eliminate NPD following positive charge flow^16^,^17^. To investigate Cl^−^ secretion or CFTR activity, either Cl^−^ -free Ringer solution or a cAMP-elevating compound was added to the perfusate^18^,^19^.

Application of NPD test to lung edema is supported by accumulating preclinical evidence. The baseline NPD of rescued *scnn1a* deficient mice was reduced by about 50% compared with wild-type mice^20^. In parallel, the rate of AFC of the rescued *scnn1a* knockout mice was approximately half that of wild-type litters^20^. However, the baseline NPD and the rate of AFC of *scnn1b* or *scnn1g* deficient mice reduced quite little than those of *scnn1a* deficient mice^21^,^22^. In contrast, gain-of-function Liddle’s mutants increased the baseline NPD and the rate of AFC^23^. Besides, AFC activated by cAMP-elevating agents was inhibited in CFTR knockout mice^24^. A marked suppression in amiloride-sensitive alveolar fluid transport was observed in injured lungs, in particularly damaged by systemic hypoxia^15^. The correlation of impaired machinery of salt and fluid transport across the alveoli and NPD was documented in several preclinical models^15^. These preclinical studies in gene-manipulating and lung injury models demonstrate a consistent functional alteration between nasal and alveolar transepithelial ion transport, and correlations between ion transport activity and their transcription/translation levels. Clinical studies suggested that transepithelial edema fluid resolution was impaired in ALI/ARDS^15^,^25^,^26^,^27^.

The aim of this study was to characterize the alterations of NPD in ALI associated with systemic hypoxia. We performed meta-analysis and correlation analysis with pooled data extracted from eleven clinical studies. As the first meta-analysis of the relationship between ion transport activity and severity of ALI, a significant decrease in both total NPD and ENaC fraction was found in HAPE and RDS patients. SpO_2_ correlated with ENaC-associated NPD in patients but not in healthy controls. Further, SpO_2_ was inversely associated with fluid secretion or CFTR activity in the respiratory epithelium. Of note, the total NPD values were not proportional to ENaC-contributed fractions. We conclude that ENaC-associated NPD is more sensitive to hypoxia associated ALI/ARDS than other fractions. A clinical trial will further confirm the O_2_ sensitivity of ENaC, which may be a proper marker of severity and prognosis of edematous lung injury.

## Results

### Characteristics of included studies

**Figure 1:**
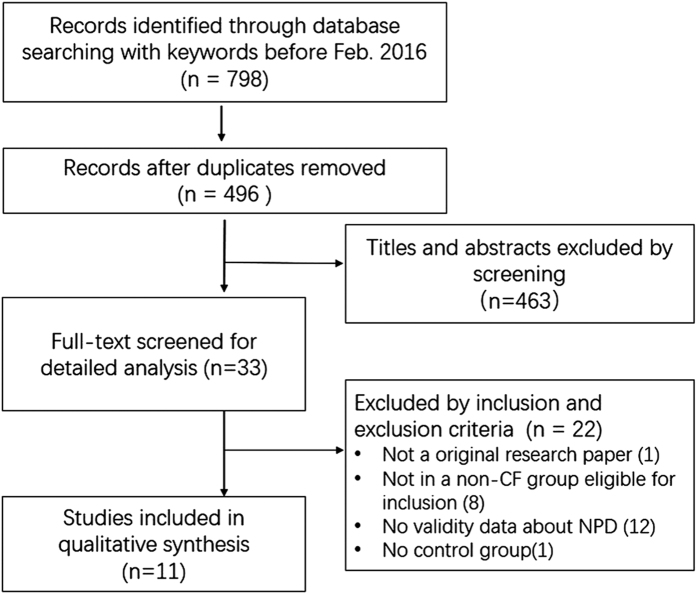
Flow diagram of the literature search and selection.

Our detailed procedure for searching and selecting studies are depicted in Fig. 1. Based on the multiple searching strategies, 798 studies were identified. Among of them, 302 studies were removed for duplicates. The remaining 496 articles were screened and 33 full-text literatures were pre-selected. Eleven case-control studies including 4 randomized, placebo-controlled and 7 nonrandomized controlled studies, from the United States, France, German, Swiss, and Finland in last two decades were finally selected per the inclusion criteria. Among these included studies, seven studies were on high altitude pulmonary edema (HAPE)^16^,^18^,^19^,^28^,^29^,^30^,^31^, and the other four studies on respiratory distress syndrome (RDS)^25^,^27^,^32^,^33^. These studies encompassed NPD data obtained with a similar recording procedure: basal level → applied amiloride (100 μM) or Na^+^ -free solution → Cl^−^ free medium and CFTR activator, isoproterenol (10 μM) (Table 1). ENaC and CFTR contributed NPD fractions were computed, respectively, as amiloride-sensitive and isoproterenol-activated net NPD fractions. The retrieved studies included 409 persons, 201 were healthy controls, and 208 were HAPE (143) and RDS patients (65). The average range of age for controls and ALI were newborn infants to 41 ± 8 yr, and newborn infant to 46.9 ± 7.8 yr, respectively. There were 218 adults (53.3%) and 191 children or infants (46.7%). Although there was a large span in age between studies, we did not see major discrepancies in the age cohort. To analyze correlations of altitude, SpO_2_, and NPD, additional data were retrieved^16^,^18^,^19^,^25^,^27^,^28^,^29^,^30^,^31^,^32^,^33^ and included in Supplementary Tables 1–3.

### Divergent basal NPD between lung injury and healthy controls

**Figure 2:**
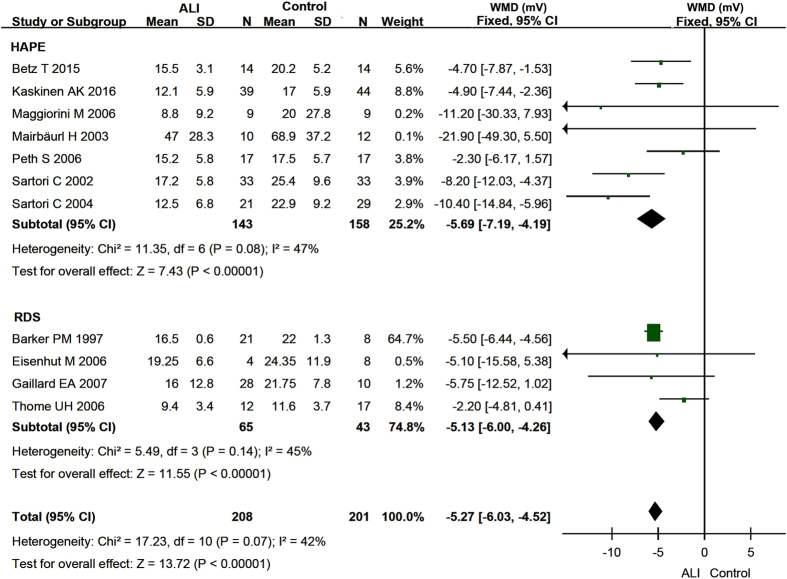
Forest plot of the Basal NPD. The green squares represent the weighted mean difference (WMD) of each study, the horizontal line represents 95% confidence intervals (95% CI), and the black diamond represents the summary of weight mean difference.

All of eleven identified studies^16^,^18^,^19^,^25^,^27^,^28^,^29^,^30^,^31^,^32^,^33^ reported basal NPD with I^2^ values of 42.0% for pooled data, 47.0% for HAPE, and 45% for RDS, indicating insignificant heterogeneity. We therefore applied the fixed-effects model for both the pooled and subgroups of basal NPD data. The meta-analysis revealed that the basal NPD values of pooled studies were significantly lesser than those of healthy control, as shown by the pooled weighted mean difference (WMD) of −5.27 mV (95% CI, −6.03 to −4.52 mV) (P < 0.00001) between two groups (Fig. 2). Moreover, a significant reduction in basal NPD level was seen for both HAPE (−5.69 mV, 95% CI: −7.19 to −4.19 mV) and RDS patients (−5.13 mV, 95% CI: −6.00 to −4.26 mV) as shown in Fig. 2 (P < 0.00001). Identical results are obtained when we further subgroup HAPE and RDS based on the experimental design (data not shown).

### ENaC-contributed NPD fraction between lung injury and healthy controls

**Figure 3:**
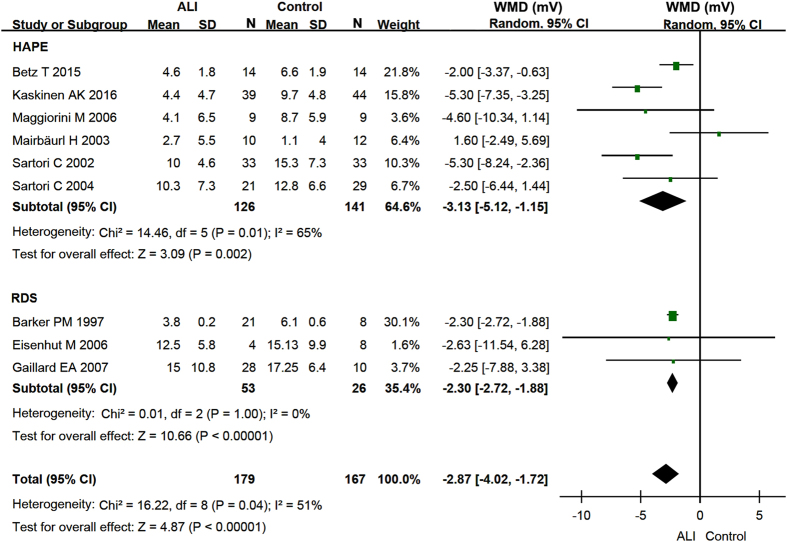
Forest plot of the ENaC-associated NPD. The green squares represent the weighted mean difference (WMD) of each study, the horizontal line represents 95% confidence intervals (95% CI), and the black diamond represents the summary of weight mean difference.

Nine studies^18^,^19^,^25^,^27^,^28^,^29^,^30^,^31^,^32^ reported the amiloride-sensitive NPD to reflect ENaC activity (Table 1). Combined ENaC-associated NPD of two types of lung injury patients substantially decreased by −2.87 mV (95% CI: −4.02 to −1.72 mV, P < 0.00001) (Fig. 3). Further, the random-effects model was used to analyze whether this decreases in ENaC function occurred in seven HAPE and two RDS studies. A marked drop in amiloride-inhibitable NPD fraction was observed by −3.13 mV (95% CI: −5.12 to −1.15 mV) in HAPE (P = 0.002) and by −2.30 mV (95% CI: −2.72 to −1.88 mV) in RDS (P < 0.00001) (Fig. 3). Identical results are obtained when we further subgroup HAPE and RDS based on the experimental design (data not shown).

### Amiloride-insensitive fraction of NPD: lung injury vs controls

**Figure 4:**
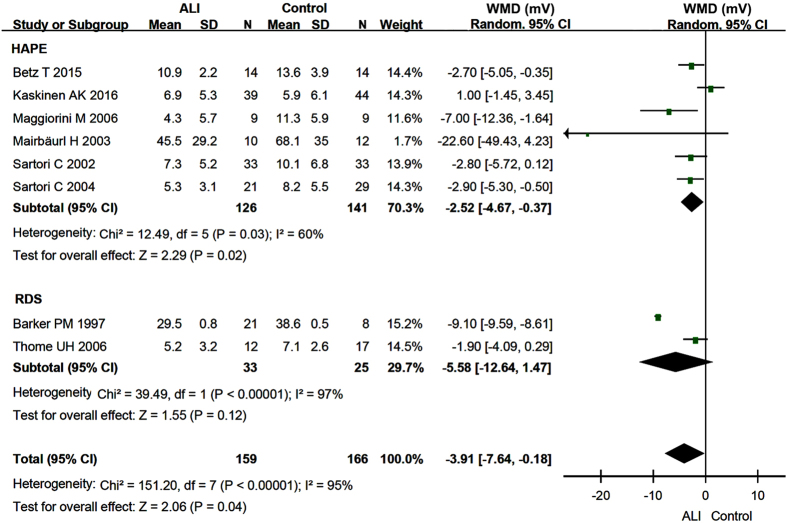
Forest plot of the amiloride-resistant NPD. The green squares represent the weighted mean difference (WMD) of each study, the horizontal line represents 95% confidence intervals (95% CI), and the black diamond represents the summary of weight mean difference.

Amiloride-insensitive (or amiloride resistant, AR) NPD was the remaining value in the presence of amiloride. It was the sum of electrogenic cation and anion transport pathways but not ENaC. Pooled AR NPD values of both HAPE and RDS patients in 8 studies^18^,^19^,^25^,^28^,^29^,^30^,^31^,^33^ were significantly reduced by −3.91 mV (95% CI: −7.64 to −0.18 mV) compared with those of controls (P = 0.04) (Fig. 4). The AR NPD values of HAPE patients decreased by −2.52 mV (95% CI: −4.67 to −0.37 mV, P = 0.02) (Fig. 4). By comparison, a doubled decrease by −5.58 mV (95% CI: −12.64 to 1.47 mV) but statistically insignificant in AR NPD of 33 RDS patients was found (P = 0.12) (Fig. 4). Identical results are obtained when we further subgroup HAPE and RDS based on the experimental design (data not shown).

### Altered expression of β ENaC and Na^+^/K^+^-ATPase of HAPE

**Figure 5:**
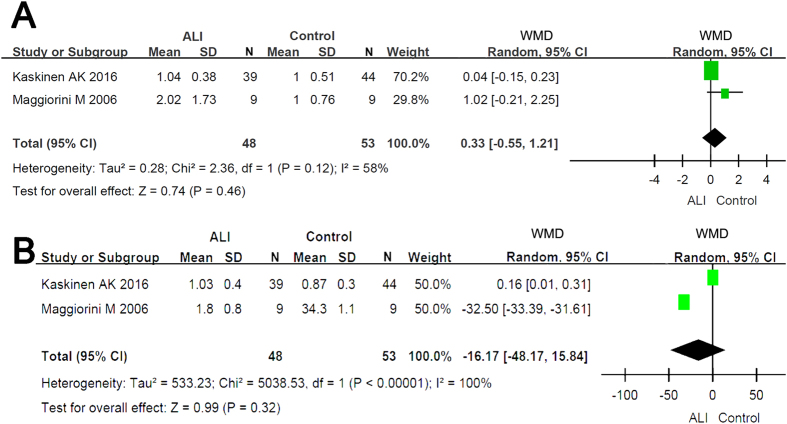
Forest plot of the expression of β ENaC (5A) and α1 subunit of Na^+^/K^+^-ATPase (5B). The green squares represent the weighted mean difference (WMD) of each study, the horizontal line represents 95% confidence intervals (95% CI), and the black diamond represents the summary of weight mean difference.

Two HAPE studies^19^,^28^ semi-quantitatively detected the transcripts of β ENaC and α1 subunit of Na^+^/K^+^-ATPase. Our results show that the slight alterations at the transcriptional level in neither β ENaC nor α1 subunit of Na^+^/K^+^-ATPase are significant (P = 0.46 for β ENaC, P = 0.32 for α1 subunit of Na^+^/K^+^-ATPase, respectively) (Fig. 5).

### Sensitivity analysis of the meta-analysis

**Figure 6:**
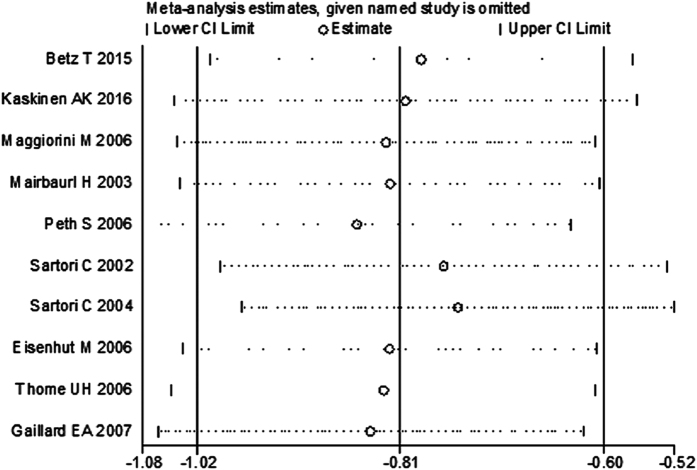
Plot of sensitivity analysis of basal NPD values between normal subjects and patients with HAPE and RDS.

To test the inclusion criteria, we omitted one study^25^, which did not report patients’ age, to test whether the sensitivity of meta-analysis. The combined SMD for other ten studies estimated by the sensitivity plot (Fig. 6) was 0.81 (−1.02, −0.59). These results indicate that in the overall meta-analysis, no single study significantly changes the combined results, and that the outcomes of meta-analysis are statistically stable and reliable.

### Publication bias of the included studies

**Figure 7:**
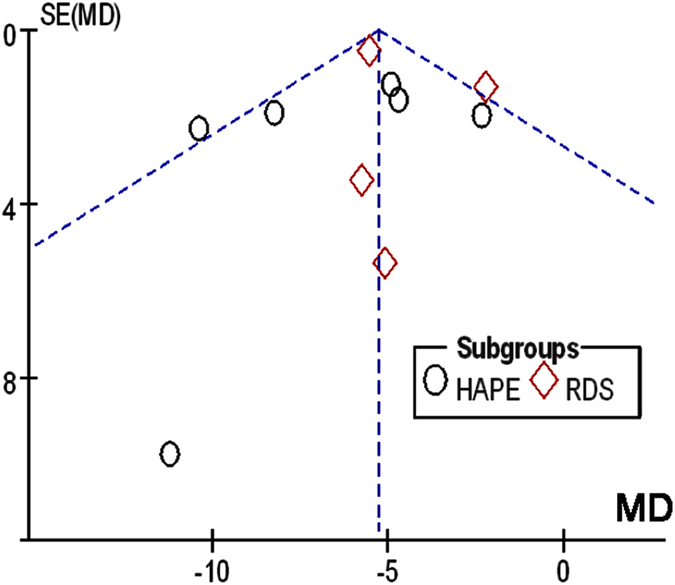
Egger’s publication bias plot of the NPD values between healthy controls and lung injury. MD, mean difference; s.e., standard error.

Given the basal NPD was analyzed by all eleven studies for both patients and normal controls, publication bias of the basal NPD values was analyzed for heterogeneity. Egger’s plot showed no substantial asymmetry (Fig. 7). Egger’s regression test shows an insignificant change in publication bias (t = −1.45, P = 0.182, > 0.05) (Table 2).

### Correlation of altitude and NPD

To estimate the role of altitude for the incidence of HAPE, we surveyed the association of altitude with other parameters (Table 3). As anticipated, altitude negatively correlated with peripheral blood oxygen saturation level (SpO_2_) (r = −0.84, P = 0.01), with a well-established SpO_2_ value of 98% at the sea level for healthy persons. Subsequently, we identified an inverse relationship between altitude and ENaC-associated NPD (r = −0.65, P = 0.01) for HAPE, and a positive association with CFTR-mediated NPD (r = 0.7, P = 0.08) for healthy controls. By comparison, these correlations are less significant statistically in the other groups. There was no association between altitude and amiloride-resistant NPD.

### Correlation of altitude-dependent SpO_2_ and NPD

Hypoxia is a hallmark of lung injury severity, whereas the effects of altitude may be contributed by hypoxia, low temperature, and exhaustion. To further analyze the contribution of hypoxia, we extracted lab data of SpO_2_ and computed from altitude, if unavailable, based on their relationship obtained in Table 3 (SpO_2_ = 98.94 – 0.006 × altitude). Given the causal relationship between pulmonary edema and SpO_2_ level, the data from patients and controls were analyzed separately (Table 4). SpO_2_ correlated with ENaC-dependent NPD fraction of HAPE (r = 0.66, P = 0.01), but not for controls. Moreover, SpO_2_ apparently showed a tendency to correlate with total, CFTR-contributed, and AR NPD fractions reversely.

### Correlation of basal NPD and fractions

Association of basal NPD and fractions associated with cation and anion transport pathways has not been reported. We analyzed their intrinsic relationships (Table 5). Our results show that there is a strong correlation between basal NPD and amiloride-insensitive NPD in both controls (r = 0.97, P = 1.1E-06) and patients (r = 0.97, P = 4.7E-09). In contrast, no significant correlation was found between basal, amiloride-sensitive, and CFTR-associated NPD values. Of note, ENaC-associated NPD might correlate with total reversely and positively, respectively, for healthy controls (r = −0.32) and HAPE (r = 0.13).

## Discussion

NPD is one of few clinical assays to examine the transalveolar fluid transport in edematous lung injury. This approach has long been used for cystic fibrosis characterized by an abnormal bioelectrical profile in the airway, however, the feasibility of NPD in alveolar injury remains uncertain. We conducted the meta-analysis of extracted NPD data from eleven published data, and found that baseline NPD in both HAPE and RDS was suppressed significantly. This reduction was partially due to eliminated ENaC activity, which is sensitive to O_2_ supply. In sharp contrast, cAMP-activated fluid secretion across nasal epithelium, predominately via CFTR was apparently augmented. Our analyses demonstrate that hypoxia associated with ALI inhibits amiloride-sensitive NPD (fluid re-absorption) and stimulates CFTR-associated NPD (chloride secretion).

**Figure 8:**
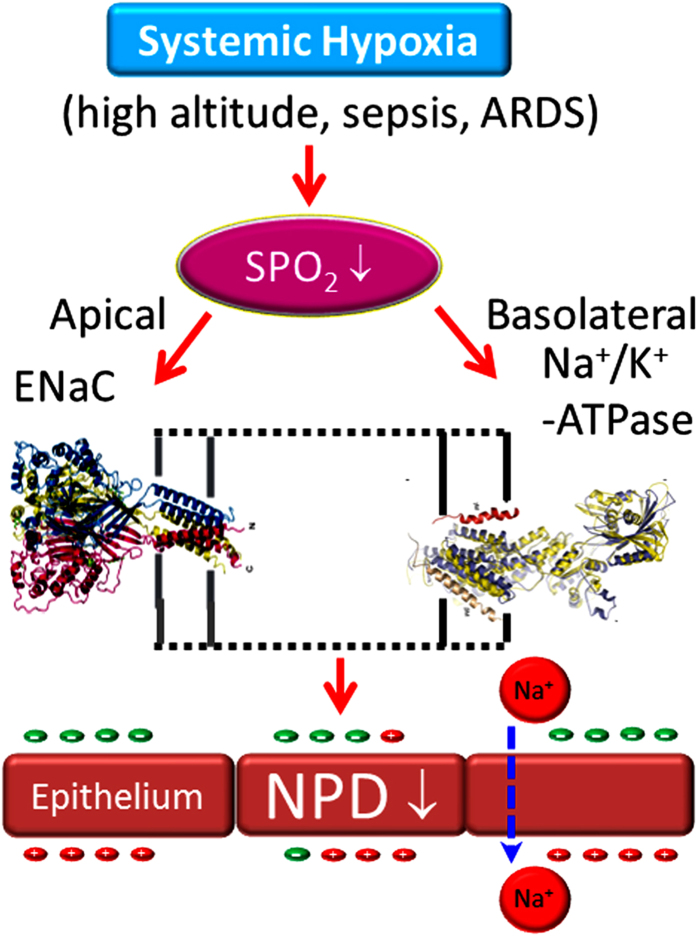
Schematic model for reduced NPD in systemic hypoxia-induced lung injury.

This systematic review suggests that total NPD could be a great indicator of impaired AFC in HAPE and ARDS. In alveolar epithelial cells, both ENaC and CFTR served as critical pathways for fluid re-absorption in the air spaces^2^,^24^. Transepithelial movement of positively charged Na^+^ ions via apical ENaC and basolateral Na^+^/K^+^-ATPase hyperpolarizes the respiratory epithelium. In contrast, cAMP-activated CFTR could transport negatively charged Cl^−^ ions bi-directionally to form Cl^−^ influx or efflux across the epithelial layer. Thus, CFTR contributes to both fluid secretion and re-absorption. It is worthy to notice that alveolar CFTR removes alveolar fluid in a way similar to ENaC^24^; whereas CFTR in the airway may mainly contribute to mucus secretion^34^,^35^. Although nasal CFTR was activated by a cAMP-elevating agent in the absence of Na^+^ or presence of amiloride in these selected studies, we do not think this is the scenario of ALI. Under physiological conditions, AFC via CFTR might be injured in HAPE and ARDS. Additionally, the clinical trial for treatment of ALI/ARDS with β receptor agonists failed^36^,^37^. Taken together, we believe that the flow of positive charges via ENaC contributes to the reduced NPD significantly in HAPE and ARDS, and that CFTR-contributed NPD fraction cannot be used to reflect AFC (Fig. 8).

The basolateral Na^+^/K^+^-ATPase catalytically consumes ATP and provides driving force for Na^+^ ions to pass through the ENaC channel pores. ENaC molecules themselves were intrinsic O_2_ sensitive^38^. However, we cannot rule out the possibility that hypoxia may disrupt mitochondrial bioenergetics and metabolism. If Na^+^/K^+^-ATPase does not have enough ATP as substrates, or low O_2_ influences its capability to catalyze ATP products, amiloride-inhibitable Na^+^ transport via ENaC will show a significant decrease. On the other hand, O_2_-sensitive K^+^ channels modulated ENaC and Na^+^/K^+^-ATPase^39^,^40^. Indeed, K^+^ channel modulators altered NPD^16^. It is conceivable to reason that hypoxia inhibits K^+^ channels and subsequently leads to dysfunctional vectorial Na^+^ transport in HAPE and ARDS. Unfortunately, these included studies did not record K^+^ channel-mediated NPD.

Transcripts of ENaC subunits individually and diversely related to nasal transepithelial ion transport, and only α ENaC subunit was positively correlated with *in vivo* NPD^41^. NPD positively related with α ENaC mRNA level, reversely with β ENaC mRNA content, and unrelated with γ ENaC transcripts. Two of included eleven studies measured the expression of β ENaC subunit at the mRNA level^19^,^28^. Thus limited data were available to perform meta-analysis and to support the observation that the transcripts of β ENaC were inversely related to NPD^41^. Moreover, the analysis of α1 subunit of Na^+^/K^+^-ATPase only was reported by Maggiorini *et al*.^28^. The sample size was too small and the expression of other two critical isoforms was not available. Additional studies are needed for further analysis. Importantly, post-translational modifications of ENaC proteins are crucial in electrically detectable channel activity, e.g., proteolysis of γ ENaC proteins by urokinase^42^. Evaluation of modified ENaC proteins, for example, cleaved subunits and the status of phosphorylation, are essential to analyze the correlation of ENaC proteins and NPD.

Our analysis shows a potential reserve association between CFTR fraction of NPD and SpO_2_ or altitude for both controls and HAPE patients (Tables 3, 4, 5). In sharp contrast to ENaC, *in vivo* CFTR activity, as reflected by cAMP-elevating agent activated Cl^−^ secretion tends to be augmented by altitude-dependent hypoxia. These results are apparently inconsistent with previous studies^43^,^44^. A mild reduction in CFTR mRNA (semi-quantitative assay) in freshly excised human airway tissues of end-stage lung diseases (hypoxic) compared with normoxic donors was described^43^. Another *in vitro* study in murine and human sinonasal epithelial cells reported a significant depression in CFTR-contributed Isc level^44^. This discrepancy could be due to differences in severity of lung injury (transient HAPE vs end-stage lung diseases). It may be due to the measures functional measures (*in vivo* NPD) of ion transport across nasal epithelium and total mRNA in excised airway specimens. The mRNA level has various relationships with the function of ENaC proteins *in vivo*, that is, amiloride-sensitive NPD^41^. This may be the scenario of *in vivo* CFTR activity and its transcriptional level. The differences may also result from low temperature at the high altitude. CFTR expression and activity are temperature-dependent^45^,^46^, and the responses of CFTR and ENaC to low temperature are opposite^47^. Our data retrieved from human studies are supported by these *in vitro* studies. Whether exhaustion of mountain climbers contributes to the diverse observations remains obscure. In HAPE lungs, increased fluid secretion through CFTR combined with reduced re-absorption via ENaC may be a novel pathogenic mechanism for lung edema caused by hypoxia and low temperature (Fig. 8)

### Limitations

Some limitations of the study should be taken into consideration when interpreting the results of meta-analysis and correlation analysis. First, heterogeneity was observed in the ENaC-contributed and amiloride-resistant NPD fractions in striking contrast to the pooled basal NPD values. The diversity is most likely due to the reduced sample size. Second, the number of patients and healthy controls was relatively small in each included study. Therefore, a much larger sample size from different ethnic populations is required for future analysis. Third, since the ethnic origins of patients and healthy controls were Caucasian, the results shall be confirmed in other ethnic populations, including Asian, African, Hispanic Americans, and Latino Americans. Finally, our findings cannot be confirmed by the expression of all of four ENaC subunit proteins (α, β, δ, γ) and two Na^+^/K^+^-ATPase subunits (α and β) and FXYD proteins due to lack of sufficient original data. Therefore, the results of this study are limited to the sum of transapical and transbasolateral Na^+^ transport, and cannot be linked to underlying cellular and molecular basis^15^.

### Conclusion

In summary, the present review demonstrates that the decrease in both total NPD and ENaC-associated NPD fraction may be a reflection of impaired edema fluid resolution in HAPE and RDS patients, and that nasal ENaC activity positively correlates with the severity of lung edema, as shown by SpO_2_ level. The total NPD values may not be proportional to the SpO_2_ level, as another NPD fraction associated with CFTR is activated by hypoxia. Conclusively, the ENaC fraction of NPD is feasible to predict the fluid resolution in the air sacs. Our findings provide fundamental data for further clinical trials to evaluate edema fluid resolution in HAPE and ARDS with bedside NPD assay, and for mechanistic preclinical studies to examine the effects of hypoxia and inflammatome on the homogeneity of transepithelial ion transport between nasal and alveolar epithelium.

## Methods

We conducted our systematic review in accordance with the methods recommended in the PRISMA guidelines.

### Literature Search

Four independent investigators searched the potential studies in NCBI PubMed, EMBASE, Scopus, Web of Science, Ovid MEDLINE, and Google Scholar before February 2016, using the search strategy: (nasal AND potential difference OR NPD) AND (pulmonary edema OR lung injury OR respiratory distress syndrome OR sepsis OR asthma OR bronchitis OR bronchiectasis OR ENaC OR amiloride OR epithelial OR SCNN1a OR SCNN1b OR SCNN1g OR SCNN1d). Our search was not limited by language, and year and type of publications. Subsequently, we extended our search using (ARDS OR ALI OR RDS OR HAPE) AND (NPD or nasal transepithelial potential difference). When the abstracts of related studies were reviewed and selected, two functions of NCBI PubMed, “Similar articles” and “Cited by PubMed Central articles”, were activated to find additional studies. We collected data from those studies with fully published articles available, not from any conference abstracts. The hits from the aforementioned databases of individual reviewer were finally pooled.

### Inclusion Criteria

All eligible studies meet the following criteria: (1) the species was human or humans; (2) the publications were original studies with case-control design, but not review or editorials or commentary; (3) the objectives of at least one group were HAPE and RDS with healthy controls; (4) the data were collected *in vivo* but not *in vitro* or *ex vivo*; (5) there were no statistically significant differences in gender, age, race between control and case groups; and (6) results were expressed or can be converted or digitized to mean ± SD.

### Exclusion Criteria

Studies were excluded if they were conference abstracts, case reports, or review articles. The studies of cystic fibrosis or patients with identified CFTR mutants were not included. Studies of allergic rhinitis^48^, asthma^49^,^50^,^51^, bronchiectasis^52^,^53^,^54^, and COPD^55^ were excluded due to significant heterogeneity, less than two studies to perform meta-analysis, or diversity in recording procedures for NPD. Of note, these three airway diseases are not characterized by systemic hypoxia and are local dysfunctions of the airway, but not the lung.

### Data Extraction

The selected studies used a similar procedure to record NPD. Basal NPD value was obtained with physiological saline (i.e., Ringer’s solution), followed by application of amiloride (100 μM) or Na^+^ free solution to inhibit ENaC, low or Cl^−^ free perfusates to eliminate Cl^−^ transport, and finally a cAMP-elevating agent, isoproterenol (10 μM) to detect CFTR-mediated Cl^−^ secretion. Original data (mean ± SD) of basal (or maximal or total) NPD, NPD values at the presence of amiloride (AR) or Na^+^-free Ringer solution, application of isoproterenol in low Cl^−^ or Cl^−^ free perfusates, computed NPD fractions for ENaC and CFTR were retrieved for both healthy controls and patients. If the disease group was further classified into mild, moderate, and severe, the data for severe subjects was collected. For the studies representing NPD values as median and interquartile range^19^, we converted the data to SD using the formula of SD = (Q75 − Q25)/1.35, when n > 25, the median can be used to estimate mean directly^56^. If the data was represented by mean and 95% confident interval (CI), then 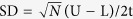, where t is calculated as the function of *tinv* (0.05, N-1)^56^, N is the sample size, U is the upper limit, L is the lower limit. If the data was showed by median and range^27^,^32^, then mean = (a + 2m + b)/4, and SD^2^ = 1/12 ((a − 2m + b)^2^/4 + (b − a)^2^), where m is median, a and b are lower and upper of the range, respectively^57^. For studies representing data as plots or graphs^18^,^29^, we retrieved data using GetData Graph Digitizer, v2.26 (http://getdata-graph-digitizer.com/).

### Statistical Analysis

We pooled weighted mean differences (WMDs) of NPD values, altitude, SpO_2_, ENaC and Na^+^/K^+^-ATPase expression level from eligible studies, which were identified with 95% confidence intervals (95% CIs). Heterogeneity of extracted data was assessed using the Cochran’s Q statistic as the P-value and I-square statistic (I^2^) in the pooled analyses, representing the percentage of total variation across studies^58^. If the P-value was less than 0.05, or the I^2^-value was greater than 50%, the summary estimate was analyzed in a random-effects model. Otherwise, a fixed-effects model was applied. Bias between selected studies was detected using the visual symmetry of funnel plots^58^. Asymmetrical distribution of data suggests possible publication bias. Publication bias was assessed by observing the symmetry of funnel plots with the Begg’s adjusted rank correlation test and Egger’s test^59^. The stability of the results was confirmed by sensitivity analysis. Key finding of meta-analysis using both STATA v.11 (StatCorp. College Station, TX) and Review Manager (RevMan) for Window, version 5.3 (Copenhagen: The Nordic Cochrane Centre, The Cochrane Collaboration, 2014) were confirmed by another independent investigator with program R^60^.

### Correlation Analysis

Correlation analyses were carried out using R software, the correlation coefficient was computed based on t test evaluation after Pearson’s product-moment. As SpO_2_ is negatively correlated with altitude^61^, it was considered as a positive control, the correlation co-efficient and P value of which are criteria for significance.

## Additional Information

**How to cite this article**: Su, Z. *et al*. Systematic review and meta-analysis of nasal potential difference in hypoxia-induced lung injury. *Sci. Rep.* **6**, 30780; doi: 10.1038/srep30780 (2016).

## Supplementary Material

Supplementary Information

## Figures and Tables

**Table 1:** Data extracted from selected studies.

Study	Diagnosis (N)	Age (year)	Basal NPD (mV)	ENaC NPD (mV)	AR NPD (mV)	β ENaC	α1 Na^+^/K^+^- ATPase
Betz T 2015^18^, QRCT	Control (14)	38.5 ± 8.3	20.2 ± 5.2	6.6 ± 1.9	13.6 ± 3.9		
	HAPE (14)	46.9 ± 7.8	15.5 ± 3.1	4.6 ± 1.8	10.9 ± 2.2		
Kaskinen AK 2016^19^, QRCT	Control (44)	2.01 ± 1.8	17.0 ± 5.9*	9.7 ± 4.8*	5.9 ± 6.1*	0.78 ± 0.4*	0.87 ± 0.3*
	Hypoxia (39)	0.71 ± 0.6	12.1 ± 5.9*	4.4 ± 4.7*	6.9 ± 5.3*	0.81 ± 0.3*	1.03 ± 0.4*
Maggiorini M 2006^62^, RCT	Control (9)	41 ± 8	20 ± 27.8	8.7 ± 5.9	11.3 ± 5.9	42 ± 32	34.3 ± 1.1*
	HAPE (9)	41 ± 8	8.8 ± 9.2*	4.1 ± 6.5*	4.3 ± 5.7*	85 ± 72.9*	1.8 ± 0.8*
Mairbäurl H 2003^29^, QRCT	Control (12)	40.5 ± 8.6	68.9 ± 37.2	1.1 ± 4	68.1 ± 35		
	HAPE (10)	42.4 ± 8.4	47 ± 28.3	2.7 ± 5.5	45.5 ± 29.2		
Peth S 2006^16^, RCT	Normal (17)	20–26	17.5 ± 5.7				
	Hypoxia (17)	20–26	15.2 ± 5.8				
Sartori C 2002^30^, RCT	Control (33)	34 ± 9	25.4 ± 9.6	15.3 ± 7.3	10.1 ± 6.8		
	HAPE (33)	36 ± 8	17.2 ± 5.8	10.0 ± 4.6	7.3 ± 5.2		
Sartori C 2004^31^, QRCT	Control (29)	31 ± 6	22.9 ± 9.2	12.8 ± 6.6	8.2 ± 5.5		
	HAPE (21)	36 ± 8	12.5 ± 6.8	10.3 ± 7.3	5.3 ± 3.1		
Barker PM 1997^25^, QRCT	Control (8)		22.0 ± 1.3	6.1 ± 0.6	38.6 ± 0.5		
	RDS (21)		16.5 ± 0.6	3.8 ± 0.2	29.5 ± 0.8		
Eisenhut M 2006^32^, QRCT	Control (8)	5.64 ± 5.0	24.35 ± 11.9*	15.13 ± 9.9*			
	RDS (4)	5.33 ± 5.7	19.25 ± 6.6*	12.5 ± 5.8*			
Thome UH 2006^33^, RCT	Control (17)	0.019	11.6 ± 3.7		7.1 ± 2.6		
	RDS (12)	0.019	9.4 ± 3.4		5.2 ± 3.2		
Gaillard EA 2007^27^, QRCT	Control (10)	0.0027	21.75 ± 7.8*	17.25 ± 6.4*			
	RDS (28)	0.0027	16 ± 12.8*	15 ± 10.8*			

Data are presented as mean ± S.D. NPD, nasal potential difference; ENaC, epithelial sodium channels; AR, amiloride-resistant. HAPE, high altitude pulmonary edema; RDS, respiratory distress syndrome. ^#^citation number. N, the number of healthy controls or patients. RCT, randomized placebo-controlled trial; QRCT, quasi-nonrandomized controlled studies.

^*^Converted from median and range.

**Table 2:** Egger’s test of publication bias between selected studies.

Std eff	Coef	Std err	t	P > |t|	95% CI
Slope	−0.002	0.641	0.000	0.998	−1.451, 1.448
Bias	−2.577	1.782	−1.450	0.182	−6.608, 1.453

Std eff, standard efficiency; Coef, coefficient; Std err, standard error; t, t test; P > |t|, 2-tailed P value; CI, confident intervals.

**Table 3:** Correlations between altitude, SpO_2_, and NPD level were analyzed with the Pearson’s product-moment correlation (r) of the software R.

Altitude vs	r	P value	df
Control			
Basal NPD	0.28	0.43	8
ENaC fraction	−0.46	0.18	8
CFTR fraction	0.7	0.08	5
AR NPD	0.37	0.37	6
HAPE			
Basal NPD	−0.06	0.83	12
ENaC fraction	−0.65	0.01	12
CFTR fraction	0.74	0.26	2
AR NPD	0.2	0.51	11

The linear regression of SpO_2_ and altitude is, SpO_2_ = 98.94−0.006 × altitude. The normal SpO_2_ value of healthy persons at the sea level is 98.94%. df, degree of freedom.

**Table 4:** Correlation of altitude-dependent SpO_2_ and NPD.

SpO_2_ vs	r	P value	df
Control			
Basal NPD	−0.23	0.40	14
ENaC fraction	0.46	0.14	10
CFTR fraction	−0.60	0.15	5
AR NPD	−0.33	0.36	8
HAPE			
Basal NPD	−0.15	0.59	13
ENaC fraction	0.66	0.01	13
CFTR fraction	−0.69	0.31	2
AR NPD	−0.43	0.13	2

See Table 3 for detailed description. Some values of SpO_2_ are computed from altitude data by the linear regression of SpO_2_ and altitude (see Table 3).

**Table 5:** Correlations of basal and fractional NPD values.

Basal NPD vs	r	P value	df
Control			
ENaC fraction	−0.32	0.20	16
CFTR fraction	0.33	0.47	5
AR NPD	0.97	1.05E-06	9
HAPE			
ENaC fraction	0.13	0.56	21
CFTR fraction	0.14	0.86	2
AR NPD	0.97	4.65E-09	13
