# Supplementary material for: Systematic review and meta-analysis of nasal potential difference in hypoxia-induced lung injury
Source: Sci Rep. 2016 Aug 4;6:30780. doi: 10.1038/srep30780 (PMC4973263; doi:10.1038/srep30780)
Supplement: Supplementary Information [file srep30780-s1.pdf]

Systematic review and meta-analysis of nasal potential difference in hypoxia-induced lung injury

Zhenlei Su, Lili Zhu, Jing Wu, Runzhen Zhao, Hong-Long Ji

Supplementary Table 1. Data for analyzing correlations with altitude or SpO_2. ENaC, epithelial sodium channels; SpO_2, blood oxygen saturation level; CFTR, cystic fibrosis transmembrane conductance regulator. * calculated from the correlation of altitude and SpO_2.

Study	SpO_2 (%)	Altitude (m)	Basal NPD (mV)	ENaC NPD (mV)	AR NPD (mV)	CFTR NPD (mV)
Betz T 2015	71.6*	4559	20.2	6.6	13.6	23.3
Betz T 2015	71.6*	4559	18.2	5.7	12.5	29.4
Betz T 2015	71.6*	4559	15.5	4.6	10.9	26.1
Kaskinen AK 2016	95.0		17.0	9.7	5.9	
Kaskinen AK 2016	90.3		13.7	6.6	5.9	
Kaskinen AK 2016	80.0		12.1	4.4	6.9	
Maggiorini M 2006	96.0*	490	20.0	8.7	11.3	
Maggiorini M 2006	71.6*	4559	8.8	4.1	4.3	
Maggiorini M 2006	96.0*	490	14.6	5.9	8.4	
Maggiorini M 2006	71.6*	4559	2.9	0.9	1.9	
Maggiorini M 2006	96*	490	16.9	6.5	10.4	
Maggiorini M 2006	71.6*	4559	4.8	1.9	2.9	
Mairbäurl H 2003	97.1	490	14.4	6.4	8.7	13
Mairbäurl H 2003	62.9	4559	27.5	3.3	24.7	38
Mairbäurl H 2003	59.5	4559	47.0	2.7	45.5	21.1
Mairbäurl H 2003	94.9	490	21	8.1	13.6	14.8
Mairbäurl H 2003	77.1	4559	31.1	2	29.2	32.6
Mairbäurl H 2003	79.2	4559	68.9	1.1	68.1	25.3
Mairbäurl H 2003	98.3*	105	17.2	3.5		12.3
Mairbäurl H 2003	72.0*	4500	21.0	3.9		14.2
Peth S 2006	99.1		17.5			
Peth S 2006	80.4		15.2			
Peth S 2006	99.1		17.5			
Peth S 2006	79.4		20.5			
Sartori C 2002	95.5*	580	17.2	10	7.3	
Sartori C 2002	95.5*	580	25.4	15.3	10.1	
Sartori C 2002	95.5*	580	27.8	14.4		
Sartori C 2004	72.0	4559	12.5	10.3	5.3	
Sartori C 2004	95.5*	580	18.0	10	8.3	
Sartori C 2004	79.0	4559	22.9	12.8	8.2	
Sartori C 2004	95.5*	580	25.6	14.8	12	

Supplementary Table 2. Data of healthy controls for analyzing correlations with basal NPD. ENaC, epithelial sodium channels; AR, amiloride-resistant; CFTR, cystic fibrosis transmembrane conductance regulator; N, the number of healthy controls.

**Study**	**Control (N)**	**Basal NPD (mV)**	**ENaC NPD (mV)**	**AR NPD (mV)**	**CFTR NPD (mV)**
Betz T 2015	14	20.2	6.6	13.6	23.3
Betz T 2015	14	18.2	5.7	12.5	29.4
Kaskinen AK 2016	44	17	9.7	5.9	
Kaskinen AK 2016	16	13.7	6.6	5.9	
Mairbäurl H 2003	12	21	8.1	13.6	14.8
Mairbäurl H 2003	12	31.1	2	29.2	32.6
Mairbäurl H 2003	12	68.9	1.1	68.1	25.3
Mairbäurl H 2003	17	17.2	3.5		12.3
Mairbäurl H 2003	17	21	3.9		14.2
Peth S 2006	17	17.5			
Peth S 2006	17	15.2			
Peth S 2006	17	17.5			
Peth S 2006	17	20.5			
Sartori C 2002	33	25.4	15.3	10.1	
Sartori C 2004	29	22.9	12.8	8.2	
Sartori C 2004	29	25.6	14.8	12	
Barker PM 1997	8	22	6.1		
Eisenhut M 2006	8	24.4	15.1		
Thome UH 2006	17	11.6		7.1	
Gaillard EA 2007	5	24.8	13		
Gaillard EA 2007	9	20	12.3		
Gaillard EA 2007	10	21.8	17.3		
Gaillard EA 2007	10	13.5	10.5		

Supplementary Table 3. Data of ALI patients for analyzing correlations with basal NPD. ENaC, epithelial sodium channels; AR, amiloride-resistant; CFTR, cystic fibrosis transmembrane conductance regulator; N, the number of patients.

**Study**	**ALI (N)**	**Basal NPD (mV)**	**ENaC NPD (mV)**	**AR NPD (mV)**	**CFTR NPD (mV)**
Betz T 2015	14	15.5	4.6	10.9	26.1
Kaskinen AK 2016	39	12.1	4.4	6.9	
Maggiorini M 2006	9	20	8.7	11.3	
Maggiorini M 2006	9	8.8	4.1	4.3	
Maggiorini M 2006	8	14.6	5.9	8.4	
Maggiorini M 2006	8	2.9	0.9	1.9	
Maggiorini M 2006	10	16.9	6.5	10.4	
Maggiorini M 2006	10	4.8	1.9	2.9	
Mairbäurl H 2003	10	14.4	6.4	8.7	13
Mairbäurl H 2003	10	27.5	3.3	24.7	38
Mairbäurl H 2003	10	47	2.7	45.5	21.1
Sartori C 2002	33	17.2	10	7.3	
Sartori C 2002	7	27.8	14.4		
Sartori C 2004	21	12.5	10.3	5.3	
Sartori C 2004	21	18	10	8.3	
Barker PM 1997	21	16.5	3.8		
Barker PM 1997	11	19.5	4.6		
Barker PM 1997	12	21.5	6.1		
Eisenhut M 2006	4	19.25	12.5		
Thome UH 2006	12	9.4		5.2	
Gaillard EA 2007	14	15.75	9.5		
Gaillard EA 2007	24	20.5	14		
Gaillard EA 2007	30	16	15		
Gaillard EA 2007	27	15	11		
